# The Nursing Supplement

**Published:** 1889-11-16

**Authors:** 


### The Nursing Mirror.

All Contributions for this Supplement should be addressed to the Editor, The Hospital, 139-140, Salisbury Court, Fleet Street, London, E.C., and should have the word "Nursing" plainly written in left-hand top corner of the envelope.

### En Passant.

Registration of Midwives.—So many good people are afraid of Parliamentary action in favour of the registration of midwives, on the ground that such an enactment will interfere with neighbourly action, that it may be well to remind them and others of the true meaning of the word. It implies services rendered for payment alone, as the following extract from an entomological dictionary shows: "Midwife, mid'wīf, n., lit., a woman who acts for a meed or reward, a woman who assists others in childbirth; pl., midwives (mid'wīvz). [A.S., mead, med, reward, and wif, woman.]

Exhibition of Nursing Uniforms.—We would remind our readers that the dolls for this exhibition must be sent to the Editor of The Hospital, The Lodge, Porchester-square, London, W., between November 24th and 30th. By kind permission of Alderman Stone, treasurer, the exhibition will be held at St. Thomas's Hospital, Albert Embankment. The judges will be Miss Gordon, matron of Charing Cross Hospital; Miss Phillipa Hicks, matron of the Hospital for Sick Children, and the Editor. There have been 94 entries for the show, but still there are one or two hospitals not represented. An Aberdeen nurse wrote to us: "I am too late to enter for a prize, but I will send a doll, as I so much wish our institution to be represented." We commend this example to nurses or friends of Middlesex, Charing Cross, Great Ormond-street, and St. Bartholomew's.

A New Cap.—A lady lately undertook to act as matron *pro tem.* to a small hospital, and had to invent a cap of the simplest construction to wear, as all the washing was done in the hospital. She therefore bought 1½ yards of strong spotted muslin, and cut off two slips two inches broad the whole length of the piece. These were hemmed and edged with frilled lace. Two triangles of muslin, each side measuring about 10 inches, were then cut; the apex of both was rounded. The side opposite the apex was pleated up to form the back of the cap; one of the bands was pinned with an ornamental pin at the apex, then brought round, crossed behind, and there pinned with a second pin, the two ends hung down and formed tails. Behold here was a cap, both cheap and becoming, which was perfectly simple to wash, and which could be made up in two minutes. We recommend the notion to any institute inventing a new uniform.

Short Items.—Major A. J. Dickson has left £1,000 to his nurse, May Margetts, and £100 to his nurse, Emma Allen.—Dr. Finley, of Leith Hospital, says the number of patients have increased since lady students attended the hospital.—Miss Peter had the honour lately of showing the Marquis of Lorne over the Edinburgh Training Home for Nurses.—Princess Mary has visited the Liverpool Infirmary; she was shown round by the lady superintendent.—The Sisters of St. Vincent have opened their soup kitchens in Paris for the winter.—Miss Annesley Kenealy has been appointed a lecturer of the Health Society.—On Thursday last the Duchess of Westminster opened St. Mary's Nurses' Home, Plaistow; being the third house opened this year in connection with the work of St. Mary's Parish Nurses. Nurse Alice of this home has just recovered from scarlet fever.—The Sister Superintendent of University College Hospital writes to say that there is no religious restriction so far as the nurses there employed are concerned.

Warwick House, Leeds.—Miss Ellen Wing, late night sister at the Leeds General Infirmary, and who was for over fifteen years on the staff there, has just left the institution, and started a "Medical and Surgical Home" in the town, but still near to the scene of her old labours. Each patient may have their own doctor, and can of course have friends to see them, or even have one friend to remain with them at any time, if their medical attendant permits. The rooms are pretty and bright looking, with every modern contrivance for a properly arranged sick-room.

Then and Now.—Last November we had one or two notices about the Chichester Infirmary, which was just then suffering from the complaints of Dr. Bostock about its untrained nurses. This November we have a different tale to tell. A course of class instruction on general nursing and anatomy is being given by the new matron, Miss Wyld, to the probationers and nurses, so that no doubt in future the private nurses sent out by the institution will prove practical, thoroughly-trained women. Miss Wyld has had many years' experience with probationers, many of whom have secured good posts in the nursing field.

Notes from Oxford.—Our great university town is looking very pretty just now, though the leaves are nearly all off the Virginian creepers which hang in masses over the old colleges. The Ratcliffe Infirmary, known by name to all nurses, is a rambling old building, but the wards are very nice when you get to them. Clean white counterpanes made to fit the beds, no pins required; and neat tables of dressings, on each of which is a jar of temporary sponges made of odd bits of absorbent wool deftly done up in gauze. The probationers at the Ratcliffe Infirmary are mostly paying, £30 being the sum required for a year's training. The morning work is heavy, but all nurses and sisters have plenty of time off, and their comfort and instruction are equally cared for by the matron, Miss Lucas. Latest improvements include hot-water pipes in the women's winter garden and the enlargement of the Alexandra and Victoria wards. The Sarah Acland Memorial and Home for Nurses in Wellington-square is a rather inconvenient block of three houses, used as a medical and surgical home. The rooms look rather bare and cheerless. There are 21 nurses engaged in private nursing, and they have a very neat uniform and wear a brown veil out of doors. The district nurses—and it is this part of the work which is a memorial to the late wife of Sir Henry Acland—do most excellent work, paying nearly 14,000 visits a year. The maternity branch is also doing well, the only midwife attending 71 cases last year, in only six of which it was necessary to call in a doctor. A few miles from Oxford is the county asylum, a pretty building surrounded by nice shrubs. The attendants here are taken on inexperienced, and have to pick up their knowledge. At the end of two years, or longer, according as vacancies occur, they become charge attendants and have tails to their caps. The head attendant on the female side is Miss Martin. All lunatics on first arriving are located in the ward where Miss Martin spends most of her time; so that before they are drafted on to a permanent ward she has thoroughly studied their peculiarities. There are dances once a fortnight, the patients mixing freely on these occasions. As few unnecessary restrictions as possible are made, and the patients seem extremely comfortable, and very fond of the medical superintendent, who has been there over 30 years.

### The Management of Children.

### II.—DISEASES OF INFANCY.

ON this subject, as may well be imagined, Miss Wood is at her best. Nothing excels experience in the management of children, and Miss Wood has made the best use of her great opportunities, and noted and observed with the instinct of a true nurse. Miss Wood's "Handbook" should be in the possession of every trained nurse, or every probationer in training for nursing children; but it should not be given to young girls who have merely a sentimental longing to visit little invalids, or to play at nursing. Hospitals have become such fashionable institutions that it is not uncommon to see the latest work on medicine or nursing lying on a drawing-room table, together with the latest novel. Especially is this the case with nursing handbooks, such as that issued by the National Health Society, or the St. John Ambulance Association. But these publications are essentially elementary, whereas Miss Wood is essentially technical, and writes most obviously for the profession and not for the public. Therefore, strongly though we recommend this book to trained nurses, we recommend "The Management of Children," by "A Mother," to women in general. With regard to "night terrors," or the sudden panics which sometimes overtake children in their sleep, it is amusing to note the similarity of the remarks of the writers of the two books we are discussing: but whereas "A Mother" acknowledges in a footnote her indebtedness to Dr. West, Miss Wood, throughout the whole length of her book only once acknowledges her indebtedness to anyone; she does not even give a list of books consulted, but leaves the reader to suppose that she has chosen to write solely her own impressions. A useful hint given by "A Mother," after remarking that a child subject to night terrors should not sleep in the dark alone, is, "Having a light in the room does not do away with the necessity for a child being in some measure shaded so as to have the eyes perfectly rested during sleep; a curtain shading the light, or a screen, answer the purpose perfectly." The nervousness of children is seldom recognised as a disease, and too often punished as naughtiness; yet the prevalence of chorea, hysteria, epilepsy, convulsions, and meningitis among children prove that their sufferings in this direction are very real. It is sad to nurse a child through any of these diseases; that the man who has thought and fought his way through life should feel the stress of the battle in irritable nerves and weakened brain seems natural, but the little child should alike be unconscious of brain or body. Miss Wood very wisely remarks that it is better to make no enquiries into the daily function than to turn a child's mind too much upon them. Judicious treatment by the mother or nurse will do much to stave off those nervous affections which, if they once attack children, are so terribly hopeless. We all know how a nurse dreads a case of tubercular meningitis in a child under seven, knowing well how rare is even the best nursing successful then. A whole chapter of the "Handbook" is devoted to infantile paralysis, and here the instructions of Dr. Barlow are given in full. But is it ever any use to try and teach massage by writing? The proper clothing very warm, so that hot-water bottles are not needed; the rubbing twice daily, which is a modified form of massage and the baths, or rather douches of hot and cold salt water, are fully described. Another chapter is devoted to tabes mesenterica and tubercular peritonitis, which are bracketed together because the nursing is somewhat similar. Of course the chief point here is the feeding, and we are sorry that Miss Wood dismisses the subject of peptonised foods so summarily. There are several ways of peptonising milk, and different doctors prefer different methods; the peptonising pellets and powders are very convenient, so are some of the many preparations advertised, but if you have not these preparations, or the doctor desires some other method, it is well that the nurse should have full knowledge of the subject. A common recipe for peptonised beef-tea, such as the following, ought certainly to have been given: To one quarter of a pound of minced raw beef, entirely free from fat, add one half-pint of cold water; cook over a slow fire, with constant stirring, until it has boiled a few minutes. Then pour off the liquor, and beat or rub the meat to a paste. Put the latter into a jar with one half-pint of cold water, and pour in the liquor previously obtained. Add to this mixture 30 grains of ext. pancreatis and 20 grains sodii bicarb., shake all well together, and keep at a temperature of about 110 deg. F., stirring occasionally, for three hours. Next boil quickly, strain and serve as required." The whole subject of peptonised foods is fully and plainly dealt with in "The Management of Children," and it is no trifling matter that a nurse should understand not only how to peptonise food, but why it is done.

*(To be continued.)*

### Examination Questions.

### PRIZE ANSWER FOR OCTOBER.

"STATE what you know of the antiseptic treatment of wounds."—As far as we know at present, putrefaction is caused by the abnormal development of "germs" or "organisms," which exist more or less everywhere, and which like warmth and moisture. The antiseptic treatment of wounds means: (1) The prevention of putrefaction in them, called keeping them aseptic. (2) The sweetening of septic wounds—that is, those which have begun to putrefy. To keep wounds aseptic.—*Lotions:* Wash with certain lotions which are said to be "germ killers," and which are called antiseptic lotions. Those most in general use are: sol. perchloride of mercury—1-2,000, or 1-1,000, or 1-500; carbolic acid lotion—1-80, or 1-40 or 1-20. *Drainage:* Drain with indiarubber tubing, strands of horse hair, or, if nothing else is available, with a strip of lint, or a few linen threads knotted together. Drains are always steeped in antiseptic lotion before being used. They serve for the free escape of moisture, and keep the wound dry. *Dressings:* Protect from all contact with the air, dust, etc., by covering with antiseptic dressings; these are made of materials that not only serve to absorb discharge from the wound, but do not admit of the passage of germs. Such are gauze prepared with corrosive sublimate, called sublimated gauze, salicylic wool, sublimated lint, boracic lint, green protective, which is soaked in antiseptic lotion before being placed over a wound—the dressing being usually kept in position by a sublimated bandage. Every surgeon has his own fancies as to the sort of dressings and lotions to be employed. *Aseptic Wounds:* Remembering that germs are everywhere, and that the contact of a second may infect a wound, a nurse should never dress a wound without: (1) Washing her hands and brushing her nails. (2) Dipping her hands in an antiseptic lotion. (3) Keeping the neighbourhood of the wound, as well as the wound itself, free from contact with everything septic, by means of a carbolised towel under, or round it, as the case may be. (4) By not keeping the wound exposed a moment longer than is necessary, covering with lint or wool, soaked in lotion, should any delay occur during the dressing of it. *Operations:* In helping a surgeon at an operation the nurse should remember that everything he requires, such as instruments, sutures, needles, etc., must be steeped in antiseptic lotion (usually carbolic acid lotion), and that if they have been used and put down on anything which has not been carbolised, they must be redipped in lotion before being handed to him again. If sponges are used, they must be wrung out in lotion after being washed. After a wound has been dressed, the nurse must watch for the least speck of blood, or discharge, that may come through the bandage, as this is said to make a sort of passage through which germs can penetrate; should the spot be trifling, it is sufficient to cover it with antiseptic wool or gauze until the visit of the surgeon, who must otherwise be sent for. *Septic Wounds:* To render septic wounds aseptic is a troublesome and often an impossible task. It is necessary (1) to try and remove any dirt or other foreign body which, by keeping up irritation in the wound, favours discharge; (2) to cleanse thoroughly, by washing out with antiseptic lotions; (3) to establish drainage, for the free escape of discharge; (4) to pursue the same system of dressing as that employed for aseptic wounds. *Iodoform:* Iodoform powder dusted into a septic wound has an antiseptic influence, by tending to lessen the discharge and detach sloughs. Besides which it, in some measure, overpowers the smell of septic wounds—perhaps the most intolerable of all the evil smells that doctors and nurses have to endure.

### Everybody's Opinion.

*[Correspondence on all subjects is invited, but we cannot in any way be responsible for the opinions expressed by our correspondents. No communications can be entertained if the name and address of the correspondent is not given, or unless one side of the paper only be written on.]*

### LADIES AS DISTRICT NURSES.

A Member of the Committee writes as follows: Several letters have recently appeared in The Hospital on "Ladies as District Nurses." In one of these, allusion is made to the East London Nursing Society, and it is implied that a new departure is being taken by the matron in charge of the Bethnal Green division, in having associated with her a lady as a district nurse. Having been on the committee of that society since its foundation, in 1868, I am in a position to speak from personal knowledge. The introduction of a lady nurse is not a novelty; we have had four lady nurses, and two are at the present moment working on our staff as resident district nurses, and highly valued by us. The system adopted by the East London Nursing Society is that of residence by the nurse in her district, and superintendence by a lady matron who has other gifts and qualifications beyond that of skilled nursing. Our twenty-four nurses are superintended by four matrons. We find that this system lends itself readily to the selection of well-principled, steady, active, homely women who have been trained in first-class hospitals. The furnished lodgings provided by the districts in which they nurse have to them the charm of a little home of their own. They adorn them with their pictures and household ornaments, and they usually have friends of their own living within reach. The continual intercourse with the lady matron and with the assistant lady, who provides nourishment and comforts for their patients, tends to elevate and refine them. After long experience, we can speak thankfully of the excellent women whom we have found to fill these posts, some of whom have worked with us for ten, or twelve, or fourteen years, making themselves loved and valued by the poor in their own locality. While we can obtain such women, we surely do well to employ them as district nurses. There is no doubt that women of better education and higher social position can nurse equally well, if not better; and whatever gifts of tact and gentleness, of tenderness and knowledge, they may bring to their work, will make that work of higher value; but it is an open question whether the health and spirits of those more gently nurtured will prove equal to the strain, and whether they are not likely to suffer from a life of isolation from their friends. The position becomes one of additional difficulty in the case of the young lady nurse. Indeed, so fully have our committee recognised this difficulty, that they have been careful to ascertain that the lady nurses who belong to their staff are living with friends or fellow-workers. This is the case with the district nurse residing with Miss Brancker, in Bethnal Green. When we began our work, more than twenty years ago, there was no question about having lady nurses to undertake it, for it would have been impossible to find them. Now we have a body of women from both classes whose efficient training, intelligence, skill, and devotion to their work is increasing year by year. Those who see both kinds of nurses at work can hardly imagine how any jealousy can exist between them. There is work for all, and they alike succeed in winning the gratitude of their patients by their skill and unwearying kindness.

### DISTRICT NURSING.

A District Superintendent writes: I should be much obliged if you would open up in your columns a discussion among superintendents of district nursing as to the best method of reaching cases of illness among the poor. We have tried here to work loyally with the doctors, taking no cases except those they send us; but after three years' trial we find they will not even take the trouble to tell us on a postcard (supplied and addressed by us) when a case requires nursing. We cannot understand how this is, and would be glad to know how other parishes and districts are worked.—[From personal experience the Editor knows that it is best when the patients themselves send for the nurse, and when there are as few formalities in securing her services as possible. The nurse must be a very tolerant woman and the friend of the patients. Such a nurse is never left idle, but always much sought. To wait for the doctors to send for the nurse is to lose the opportunity of doing great good. The doctor will be glad enough to find the nurse there, especially if she is careful to leave a written statement for him, and to fulfil his directions with exactitude.]

### THE SCANDAL AT HOPE INFIRMARY.

Justice writes: With regard to the last of the long list of scandals at Hope Infirmary, I mean placing rats in the late matron's bed, I should like to ask a few questions. I understand that the guardians, supposing it was the work of one of the lunatic patients, withdrew, absolutely, the notice issued to the officials, nurses, and servants. Granted, for the moment, that it was an imbecile as stated, in the first place I would ask, Is no one responsible for the proper custody of the lunatic inmates? If the attendants are responsible for them, how is it that they are accounted free from blame, when it must have been an act of culpable neglect on their part to allow her escape? The infirmary rules have it "That patients are not to be sent from one pavilion to another, and about the hospital, unless accompanied by a nurse," and if they may not be sent, most surely they may not be allowed to wander at will; above all, lunatics, whose wards, I believe, are supposed to be always kept locked. Are the rules made to be obeyed or disregarded as a nurse chooses? Secondly, even if the lunatic could have made her way through the administrative block and up the stairs, carrying a nest of twelve young rats, without being seen and exciting suspicion, is it, in the least degree, probable that from all the rooms, and they are numerous, she could have singled out the matron's without being discovered unless she were well directed to it? (that it was known to be the matron's is clearly stated.) I am perfectly well acquainted with the internal arrangements of the infirmary, having had the misfortune to pass a few weeks within its walls in the capacity of probationer, and I know that it is very difficult to find any special room until one is accustomed to the place. That the patient may have been there as the tool and scapegoat of another person is more difficult to deny, but when one considers that patients are all in bed at 8 p.m., and the rats were not discovered till about 2 a.m., does it not rather point to someone who was well acquainted with the home block, and who had free access to it at a later hour? Who circulated the report that it was a lunatic, and can the persons who did so prove it, fully, beyond a shadow of a doubt? If not, is it not possible that they themselves are guilty of the offence? I must ask your forbearance in taking up so much of your valuable space, but, to say the least, it seems so unaccountable that none of these things should have been thought of; and the story of the lunatic seems to me (and to others) only a convenient invention of the real offenders, brought forward to extricate themselves from the consequences of their act. I have been a subscriber to The Hospital for a little more than a year, and like it better every week. I think it is a most useful paper, and sincerely wish it every success.

### ASYLUM ATTENDANTS.

S. M. writes: Why are our Lunacy Commissioners so blind, and how long is this system of mental nursing to continue? "J. B." has spoken words so true, I could not imagine they are destined to meet with one fair-minded nurse who will contradict them. There may be some asylums—exceptions, perhaps—but I wish to speak of the average county asylum. The doctors are kind, as a rule, and I consider any woman making choice of nursing as a profession could not do much better than enter one, providing insanity was not objectionable to her. But there should be a trained nurse as matron, and let our head attendant be certificated as mental nurse, and surgical or medical. At present, a girl of twenty may, after a few months' experience, be appointed head attendant simply because she is a favourite. We want good teachers. The doctors can alter the present state of affairs in all asylums. We want a good library, useful books, lectures, and, when thoroughly trained, after three years to receive a certificate.

### INFANTINE MORTALITY.

Nurse Harris writes: Thinking the following case worth publishing in the interests of the babies, I submit facts to your notice. I was lately called suddenly at night to a five weeks' old baby, so ill that three doctors were in attendance, one being a physician of eminent authority. The infant was reduced to skin and bone, the face resembling an old man's, rather than a new arrival in this troubled world of ours; he was worn with abdominal pains, and green griping diarrhœa. It was too evident that the "bottles," composed of the usual allowance of milk and water, passed through the little sufferer, but failed to nourish him. Stupors occurred at intervals, lasting several hours, brandy and beef-tea, dropped from a sponge, kept the tiny flame flickering, but produced no marks of consciousness to cheer the watchers. Isinglass and milk proving futile, my orders were to try feeding on white wine whey This failed to help us. The infant cried piteously when able to do so; the griping, diarrhœa, and stupors continued. No time was to be lost. While gathering from the mother the history of her previous confinements, I learnt that one of her children, at six months, fell into a similar condition; all milk food was rejected, but a patent food did her good. Upon enquiring, I found the doctor had been asked if the same nourishment might be tried in this case, but he naturally hesitated at such an early age. On his next visit we renewed the request, the child was evidently going quickly; it would, at least, be a satisfaction to try. After pointing out that what nourished at six months might kill at five weeks, he gave a reluctant assent to what could only be called an experiment. Within twenty-four hours the food administered from a little sponge had these effects: the griping and sickness ceased, the diarrhœa was less, and not so offensive. At forty-eight hours the infant commenced feeble sucking, and subsequently recovered.

### Appointments.

[It is requested that successful candidates will send a copy of their applications and testimonials, with date of election, to The Editor, The Lodge, Porchester-square, W.]

North Shields Infirmary.—Miss Mary Benning, who trained at the London Hospital and held the post of staff-nurse there, has been appointed matron of the North Shields Infirmary.

Atcham Union.—Miss Tomlinson, who trained at Crumpsall Infirmary and holds the Obstetrical Societies' diploma, has been appointed head nurse at Atcham.

Royal Hospital for Children and Women.—Miss Mary Mocatta has just been appointed matron of this hospital in the Waterloo-bridge-road. Miss Mocatta trained and worked for nearly four years at University College, and was for one year sister at Charing Cross. For the past seven months she has been in charge of the nursing at the Royal Hospital for Children and Women, and has done such good work she has been permanently appointed matron. This is the first time the nursing and housekeeping appointments of this institution have been under a trained nurse.

### Notice.

Gateshead Hospital for Sick Children.—The vacancy for lady probationer has been filled up, and the matron finds it impossible to answer by post all the applications she received.

### Notes and Queries.

To Correspondents.—1. Questions may be written on post-cards. 2. Advertisements in disguise are inadmissible. 3. In answering a query please quote the number. 4. If a private answer is desired, a stamped addressed envelope must be forwarded.

Notice.—We must really ask our correspondents to be more particular in enclosing name and address. Constantly we receive queries or letters with no name attached.

### Queries.

(21) *National Aid Society.*—Where can all particulars be obtained? *A. C. C.*

(22) *Home Wanted.*—Is there anyone could tell me of any home for a poor boy of seven? His mother is dead and his father is a bad man. *Anon.*

(23) *Addresses Wanted.*—Of one or two institutions where the nurses pay a commission and receive their own fees. *F. C. H.*

### Answers.

*Miss D.* and *Miss M.*—The Chichester Infirmary. The premium is 10 guineas for a year's training, and a certificate is given.

*Enquirer E. S.* will find all her numerous questions answered in the article "How to Become a Nurse," which appeared in The Hospital for August 31st.

*A Mother.*—Do not fear for your daughter; her work will absorb all her energies, and it will be seldom indeed that she will ask leave to go to a theatre. Nurses are women, and ought to be capable of taking care of themselves. We should be the very last to complain of the fact that they are sometimes allowed late leave in order to go to a theatre.

(7) *Nursing Charts.*—From Lambert and Co., Newcastle-on-Tyne. They are only suitable for severe cases, the large sheet only taking 12 hours' nursing. *Verona.*

*Nurse P.*—We do not print queries asking for cures.

### Presentations.

Miss Wing, late night sister at Leeds Infirmary, has been presented with two library chairs and a writing table by the medical staff; with a marble clock and a purse of money by the nursing staff; with a walnut table by the lady superintendent; and an illuminated testimonial from the board of management. Miss Wing is leaving the infirmary in order to start a private nursing home.

### Vacancies.

The following vacancies are announced:

### Matrons.

Dr. Walter's Hospital for Women, £36.
Monsall Fever Hospital, £100.
North Cambs. Cottage Hospital, £40.

### Sister.

South Devon Hospital.

### Nurses.

Bath Royal United Hospital, £25.
Bedford Nurses' Association.
Bedfordshire Institute, £26.
Birmingham Fever Hospital.
Blackheath Institute, £20.
Bolton Nurses' Home.
Brixton Institute.
Bromley and Beckenham Hospital, £25; assistant, £18.
Bury St. Edmunds Hospital, £25.
Cedar Lawn Hospital, Latchford, £25.
Cheltenham Nursing Institute.
City of London Hospital, £20.
Croydon General Hospital.
Croydon Union, £20.
General Nursing Institute.
Great Yarmouth Hospital (night), £22.
Greenock Infirmary.
Herts. Infirmary, £16.
Jenny Lind Infirmary, Norwich (several), £20.
King's Cross Hospital, Dundee, £24.
Kingston Nurses' Home.
Leicester Fever Hospital.
Leicester Institution, £25.
Levick Institution, Paris.
Liverpool City Hospital, £25.
Longton Cottage Hospital (several).
Lynn Hospital, £20.
Newcastle Infectious Hospital, £30.
Northampton General Infirmary, £21.
Norwich Nurses' Home.
Nottingham Institution, £25.
Nursing Institution, 96, 97, 98, Wimpole-street, London, W., Mr. Wilson has several vacancies for thoroughly trained nurses.
Scarborough Nurses' Institute.
Sheffield Nurses' Home.
South-Eastern Hospital, S.E. (several), £30.
St. Leonards Nurses' Home (two).
Sunderland Institute.
The Wigmore Institution.
Truro Infirmary.
West Herts. Infirmary (night).
West London Hospital, £24.
Worcester Infirmary, £25.
York Home for Nurses (several).

### District Nurses.

Alverton House, Penzance, £40.
District Home, Bolton.
Hitchin Institution, £25.
Malmesbury Institute, £30.

### Probationers.

Beckett Hospital, Barnsley.
Boston Hospital.
Canterbury Hospital.
Great Yarmouth Hospital.
Truro Infirmary.

### Amusements and Relaxation.

### N.B. Third Quarterly Word Competition Commenced October 5th, 1889, ends December 28th, 1889.

Three Prizes of 15s., 10s., 5s., will be given for the largest number of words derived from the words set for dissection.

Proper names, abbreviations, foreign words, words of less than four letters, and repetitions are barred; plurals, and past and present participles of verbs, are allowed. Nuttall's *Standard* dictionary only to be used.

N.B.—Word dissections must be sent in WEEKLY, arranged alphabetically, with correct total affixed.

The word for dissection for this, the *Seventh* week of the quarter, being "PTARMIGAN."

### Results of Fifth Week

| Names. | Nov. 7th. | Totals. | Names. | Nov. 7th. | Totals. |
|---|---|---|---|---|---|
| Winifred | 82 | 672 | Sunshine | 81 | 807 |
| Adelaide | 64 | 646 | Electrician | 92 | 938 |
| Nettie Vance | 88 | 751 | Reldas | 93 | 969 |
| F. W. P. H. | 88 | 952 | H. C. | — | 562 |
| Amos | — | 596 | Rover | — | 776 |
| Reyot | — | 369 | Beryl | — | 188 |
| A. S. K. | — | 275 | Qu'appelle | 70 | 790 |
| Ellice | 94 | 992 | Hercules | — | 12 |
| Neptune | 82 | 954 | Lightowlers | 92 | 1026 |
| Ecila | 88 | 1041 | Jenny Wren | 83 | 642 |
| Tinie | — | 17 | Oakenrod | 93 | 1025 |
| Amicus | 72 | 942 | Gypsye | 90 | 871 |
| Juvenis | — | 539 | Leo | 60 | 670 |
| Sister | — | 438 | Night Watch | — | 346 |
| Jumps | 92 | 1047 | Embryo | — | 385 |
| M. W. | 90 | 992 | Africando | — | 167 |
| Esperance | 90 | 996 | Pearl | — | 299 |

### Notice to Correspondents.

N.B.—All letters referring to this page which do not arrive at 139 and 140, Salisbury-court, London, E.C., by the *first post on Thursdays*, and are not addressed PRIZE EDITOR, will in future be disqualified and disregarded.

